# Investigating the Impact of Storage Conditions on Microbial Community Composition in Soil Samples

**DOI:** 10.1371/journal.pone.0070460

**Published:** 2013-07-31

**Authors:** Benjamin E. R. Rubin, Sean M. Gibbons, Suzanne Kennedy, Jarrad Hampton-Marcell, Sarah Owens, Jack A. Gilbert

**Affiliations:** 1 Committee on Evolutionary Biology, University of Chicago, Chicago, Illinois, United States of America; 2 Department of Zoology, Field Museum of Natural History, Chicago, Illinois, United States of America; 3 Argonne National Laboratory, Argonne, Illinois, United States of America; 4 Graduate Program in Biophysical Sciences, University of Chicago, Chicago, Illinois, United States of America; 5 MO BIO Laboratories, Inc., California, United States of America; 6 Department of Ecology and Evolution, University of Chicago, Chicago, Illinois, United States of America; King Abdullah University of Science and Technology, Saudi Arabia

## Abstract

Recent advances in DNA sequencing technologies have allowed scientists to probe increasingly complex biological systems, including the diversity of bacteria in the environment. However, despite a multitude of recent studies incorporating these methods, many questions regarding how environmental samples should be collected and stored still persist. Here, we assess the impact of different soil storage conditions on microbial community composition using Illumina-based 16S rRNA V4 amplicon sequencing. Both storage time and temperature affected bacterial community composition and structure. Frozen samples maintained the highest alpha diversity and differed least in beta diversity, suggesting the utility of cold storage for maintaining consistent communities. Samples stored for intermediate times (three and seven days) had both the highest alpha diversity and the largest differences in overall beta diversity, showing the degree of community change after sample collection. These divergences notwithstanding, differences in neither storage time nor storage temperature substantially altered overall communities relative to more than 500 previously examined soil samples. These results systematically support previous studies and stress the importance of methodological consistency for accurate characterization and comparison of soil microbiological assemblages.

## Introduction

Advances in high-throughput DNA sequencing technology allow us to characterize microbial diversity in environmental samples at an unprecedented depth [1,2]. Soil microbial communities have been particularly fruitful research subjects for the application of next-generation sequencing techniques (e.g. [3,4]). However, soil is an extremely heterogeneous environment [5–7], and it is therefore essential that samples from different locations be treated in as similar a way as possible to prevent the introduction of potential biases. This highly diverse environment combined with the extreme sensitivity of modern amplicon sequencing approaches means that small differences in sample preparation can drastically alter the recovered species diversity [8,9]. Methods for extracting DNA from soil have been discussed since culture free studies of bacterial communities became possible [10–13]; however, the preservation techniques applied to the physical sample are equally important for accurate representation of these bacterial communities [14–17]. For example, both storage time [18,19] and temperature [20] drive change in community structure in human feces, sometimes affecting the recovered relative abundance of certain taxa more than others [21]. Furthermore, as interests in microbial ecology are shifting towards differences in extremely rare taxa and minor structural differences between highly similar samples (e.g. [22,23]), the importance of effective and consistent sample preservation is evident.

Lauber et al. [16] used 454 pyrosequencing to compare soil, fecal and skin samples stored at different temperatures over two weeks and concluded that variation between samples outweighed differences due to variable storage conditions. While these conclusions were promising, the sequencing depth applied to each sample was relatively low (1,304-3,022 sequences), which could lead to significant under-representation of the ‘rare’ microbial taxa in these complex communities and to misrepresentation of the relative abundance of even the more common taxa. Here, we use Illumina iTAG sequencing technology [24] to explore the impact of storage temperature and storage time on the microbiome profile of a single soil sample, observed at 5X the sequencing depth of this previous study [16] and with higher temporal resolution. Different second-generation sequencing platforms can also yield different results on community composition (e.g. [25,26]), requiring that each technology be examined separately. Illumina is increasingly being used for evaluating bacterial community composition but soil preservation techniques have yet to be explored on this platform. Our samples were analyzed with the laboratory protocols implemented by the Earth Microbiome Project (EMP), which are being used to process ~200,000 environmental samples [27–30]. We also utilize data from over 500 soil samples previously analyzed by the EMP to assess the significance of changes within the samples tested here.

## Methods

Soil was collected from the mineral soil surface to a depth of ~6 inches underneath a native riparian California bay tree at California Polytechnic State University (N 35° 18' 46.59'', W 120° 39' 7.26''). Carbon and nitrogen content were determined by dry combustion on an Elementar vario MAX CN analyzer (Hanau, Germany) and pH was determined using a 1:2 soil: water mixture with a Fisher Scientific Accumet AB15 pH meter (Pittsburgh, PA) and a Thermo Scientific Orion 8172BNWP ROSS Sure-Flow pH electrode (Waltham, MA). There was substantial organic matter buildup in this soil, but it is serpentinite in nature with low calcium and high magnesium content. The soil was thoroughly mixed to reduce the impact of patchily distributed taxa and then partitioned into 36, 0.25 g samples. Samples were stored at room temperature, 4°C, or -20°C and metagenomic DNA was extracted after one, three, seven, or 14 days following initial separation. Every sample treatment was replicated in triplicate.

16S rRNA amplification and sequencing on the Illumina MiSeq2000 were done by the EMP, following their standard protocols [24,28] and their modifications to the MO BIO PowerSoil DNA Isolation Kit procedure for extracting DNA (www.earthmicrobiome.org/emp-standard-protocols). Raw sequence data are available from NCBI’s Sequence Read Archive under study accession number SRA068971.

All 523 soil samples previously sequenced by the EMP were also used in this study. These samples were components of 12 distinct studies (EMP IDs: 632, 659, 722, 808, 1031, 1034, 1035, 1036, 1037, 1038, 1289, 1526). The identifiers of all individual samples used are given in Table S7. We downloaded the previously prepared open-reference OTU tables for each of these studies from https://github.com/EarthMicrobiomeProject/isme14.

We used QIIME [31] default parameters for quality filtering (reads truncated at first low-quality base and excluded if: (1) there were more than three consecutive low quality base calls (2), less than 75% of read length was consecutive high quality base calls (3), at least one uncalled base was present (4), more than 1.5 errors were present in the bar code (5), any Phred qualities were below three, or (6) the length was less than 75 bases). We picked OTUs using open reference UCLUST clustering against the February 4^th^, 2011 release of the Greengenes database filtered at 97% identity. Reads that did not match any sequences in the reference database at ≥ 97% identity were clustered *de novo*. We required that all OTUs have a count of at least two reads across all samples. OTUs that were not represented in the Greengenes reference tree were inserted into that tree using ParsInsert (http://parsinsert.sourceforge.net) as implemented in QIIME so that phylogenetic measures of beta diversity could be used. Evenness was calculated using the equitability metric defined in QIIME as: (Shannon entropy) / log_2_(number of observed OTUs). Alpha diversities for all samples, including those from other EMP studies, were calculated at a rarefaction depth of 6,700. For analyses that included only samples sequenced here, we rarefied our OTU table to 6,700 reads per sample for all beta diversity and supervised learning analyses and for determining the significance of particular OTU differences between treatments. We required the presence of an OTU in at least 10 samples to test it for significant correlations and differences in relative abundance between treatments.

For beta diversity analyses with previous EMP studies, we made OTU tables comparable to our own by filtering all OTUs not represented by the Greengenes reference set from all tables and merging the results. This table was then filtered for taxa represented by only a single read. Samples in this merged table were rarefied to 4,200 reads for subsequent analyses that utilized both the previously sequenced samples and the new samples.

Bray-Curtis, weighted UniFrac and unweighted UniFrac distances were used for all analyses of beta diversity. We used both analysis of similarities (ANOSIM) and distance-based redundancy analysis (db-RDA) to test for significant differences in beta diversity between treatments and used db-RDA models to evaluate the percent of variation explained by the different treatments. We also used ANOSIM and db-RDA metrics to compare just the communities comprised of rare (number of reads ≤ 10) and common (number of reads ≥ 100) OTUs. Differences in relative taxon abundance between treatments were examined using analysis of variance (ANOVA) and correlations between relative abundance and storage time were evaluated with Pearson correlations. All p-values reported for differences in taxa among treatments were FDR-corrected. All ANOSIM and db-RDA tests of significance between treatments used 10,000 permutations. We specified 10,000 trees in the forest for all supervised learning analyses.

In addition to ANOSIM and db-RDA comparisons of soil communities, we also evaluated differences between treatments by comparing the mean beta diversity distances between all samples in a particular treatment group. Lower mean beta diversities between samples show greater similarity in community composition. Therefore, these measures were useful for evaluating how consistent communities remained within treatment groups.

## Results

### Sample characteristics

The pH of the homogenized soil sample was 6.74, total nitrogen content was 0.811% and carbon content was 10.35%. Recovered sequence counts for individual sub-samples ranged from 7,239 to 18,444 (mean 12,391; Table S1). After clustering and removal of OTUs represented by single sequences, counts ranged from 6,897 to 17,850 (mean 11,752; Table S1).

### Alpha diversity

**Figure 1:**
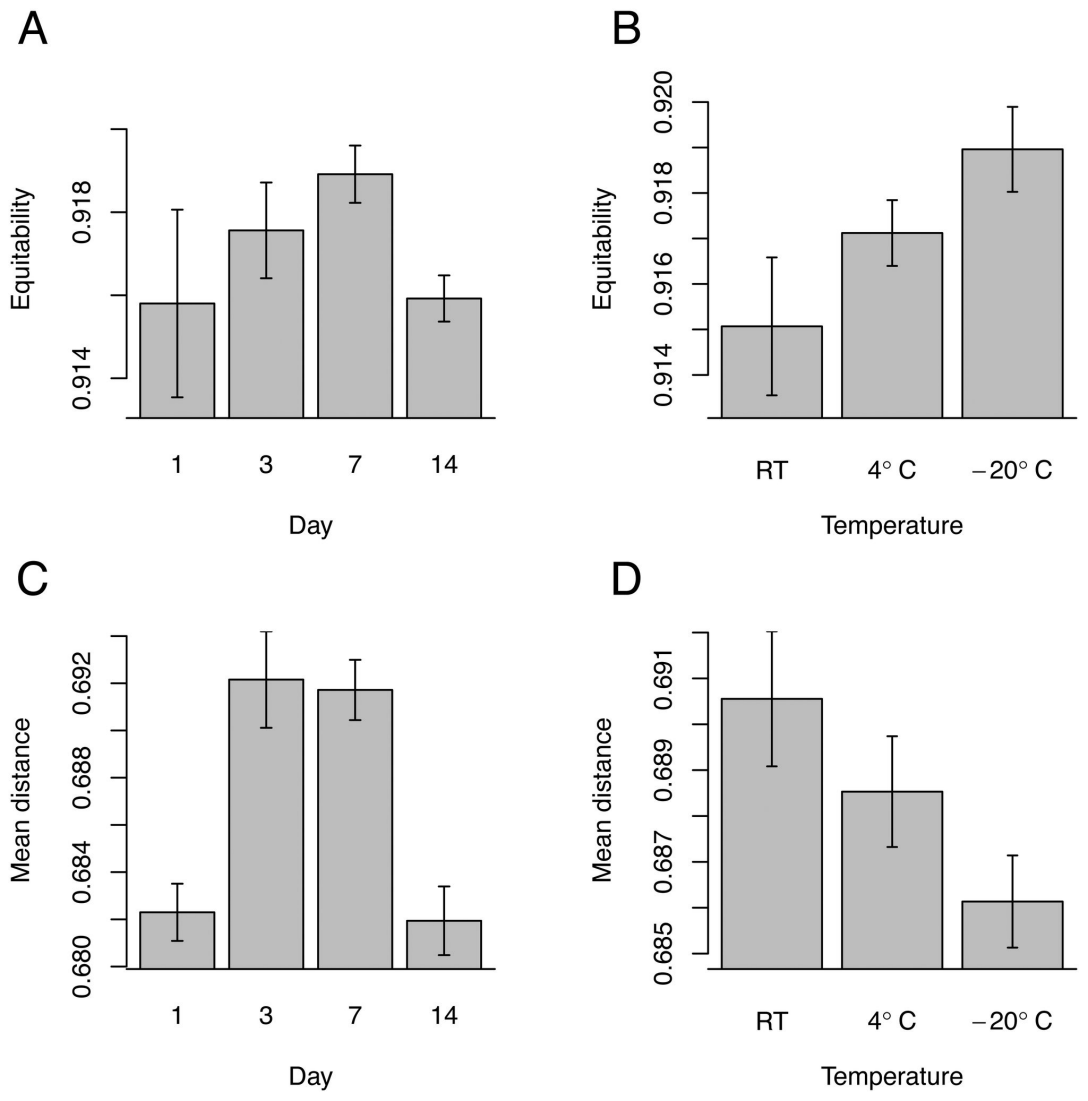
Mean ± standard error of: (A) and (B) evenness (equitability) between storage times and storage temperatures, respectively; (C) and (D) unweighted UniFrac distances between samples within each time and temperature treatment, respectively.

Samples stored at room temperature (RT) had significantly lower alpha diversity (measured as the absolute number of observed taxa (richness), evenness, and Shannon diversity) than those stored at -20°C (p < 0.0001, Table S2 Figure 1) and significantly lower evenness and Shannon diversity than those stored at 4°C (p < 0.01). Frozen (-20°C) samples had significantly greater alpha diversity than refrigerated (4°C) samples (p << 0.0001, Table S2). Samples extracted on days three and seven had significantly higher alpha diversities than those extracted on days one and 14 by all three metrics (p < 0.05). Additionally, samples extracted on day seven had significantly higher evenness than those extracted on day three (p = 0.001). The range of alpha diversities from this study was less than the ranges of alpha diversities within other individual EMP studies (Table S3).

### Beta diversity

**Figure 2:**
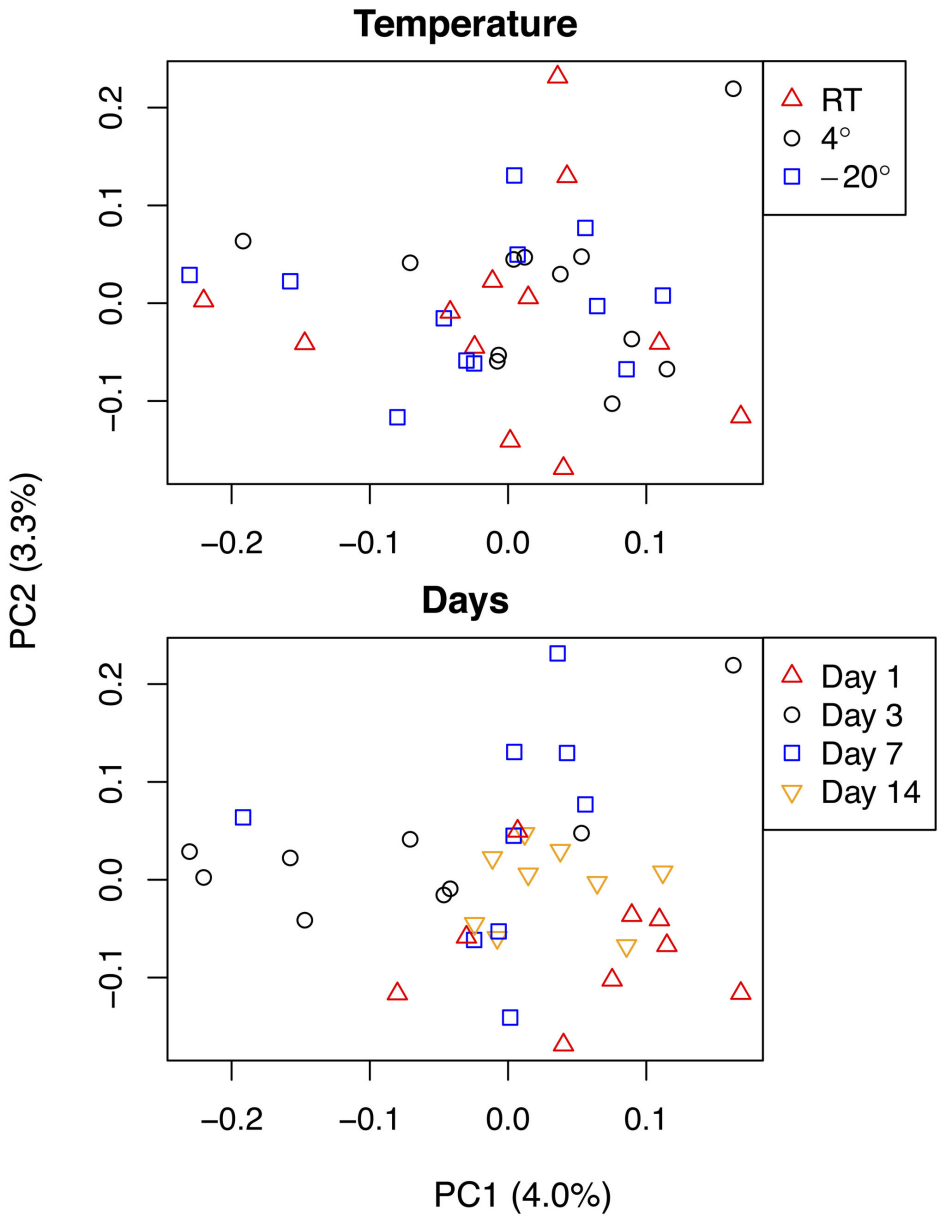
Principal coordinates plots showing similarities of samples between temperature treatments and time treatments. Pairwise unweighted UniFrac distances were used to generate plots.

Beta diversities differed significantly between storage times according to both ANOSIM and distance-based redundancy analyses using all three distance metrics (p < 0.01, Table S4 Figure 2). Time remained an influential factor when samples stored at the same temperature were compared across time points, removing the confounding effect of temperature (Table S4). Temperature was similarly analyzed without the confounding effect of time but communities significantly differed between temperatures only when stored for one day and for 14 days (Table S4). Significant differences between times held for both the rare and common communities, except that unweighted UniFrac metrics did not show significant differences in the rare community between times.

Average beta diversity distances between samples within temperature treatments were significantly lower in both 4°C and -20°C stored samples, compared with those stored at RT (T-tests, p < 0.05) for weighted UniFrac and Bray-Curtis distances (Table S5). Beta diversities within samples stored for three days and seven days were significantly higher than those stored for one and 14 days (p < 0.05, Table S5 Figure 1).

**Figure 3:**
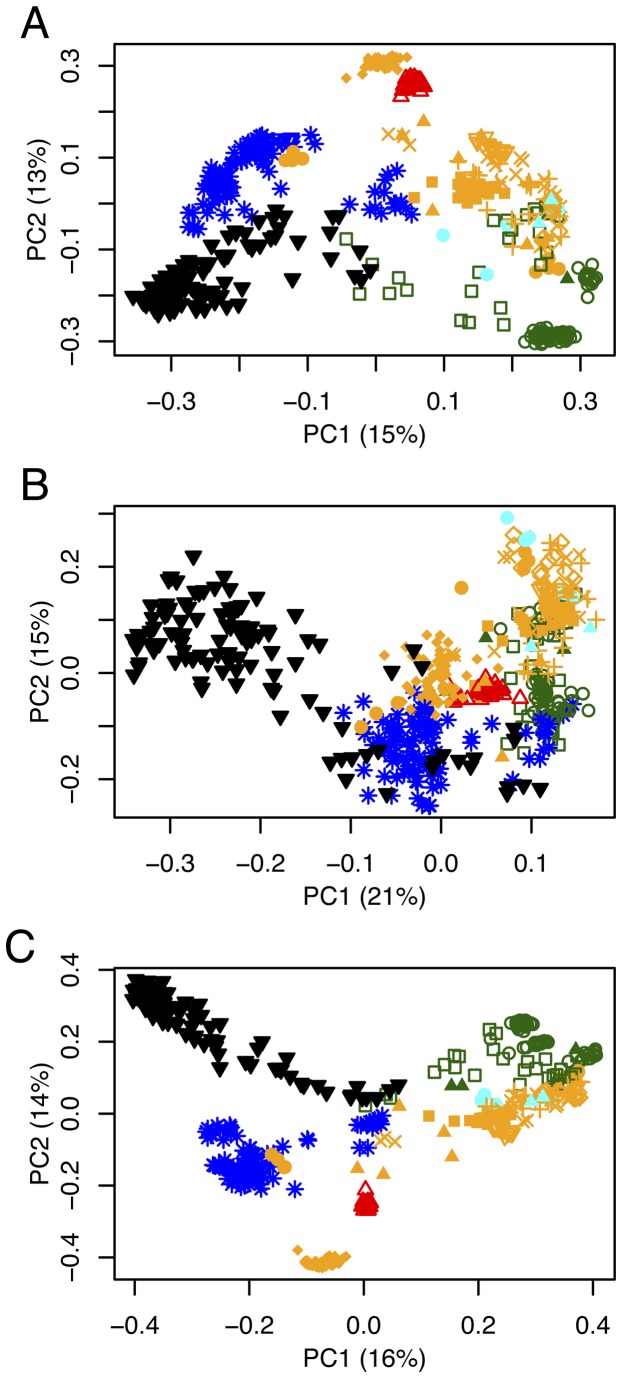
Principal coordinates plots of all Earth Microbiome Project sequenced soil samples based on unweighted UniFrac (A), weighted UniFrac (B), and Bray-Curtis (C) distances. The samples analyzed for this study are represented by open red triangles. All other samples are colored by biome: blue = polar desert, green = tundra, orange = temperate, black = warm desert, cyan = tropic. Filled triangles = EMP study 632, filled squares = 659, inverted open triangles = 722, inverted filled triangles = 1035, filled circles = 808, open diamonds = 1031, open squares = 1034, open circles = 1036, '+’s = 1037, 'X’s = 1038, and'*’s = 1526.

All communities examined for this study grouped very tightly relative to all other EMP soil samples in PCoA plots, despite the presence of other soils also collected from temperate biomes (Figure 3). Communities differed significantly between the samples analyzed here and all other EMP soils (p < 0.001, Table S4).

### Relative abundance differences by treatment

Of 2,781 OTUs tested, a single OTU in the order Actinomycetales differed significantly in relative abundance between temperature treatments (RT > 4°C > -20°C; ANOVA, FDR-corrected p = 0.000006). This OTU was relatively common, making up between 0.06% and 0.2% of the sequences in the samples compared. No OTUs differed significantly in relative abundance between time treatments. However, three bacterial families differed significantly in relative abundance between time treatments; the Rubrobacteraceae (Day 14 > Day 1 > Day 7 > Day 3; p = 0.040), the Planococcaceae (Day 1 > Day 7 > Day 14 > Day 3; p = 0.027), and the Bradyrhizobiaceae (Day 14 > Day 7 > Day 1 > Day 3; p = 0.045). The Patulibacteraceae was the only family to significantly differ in relative abundances between temperature treatments (RT > 4°C > -20°C; p = 0.039). Of the bacterial orders present, Rhizobiales (Day 14 > Day 7 > Day 1 > Day 3; p = 0.020), Rubrobacterales (Day 14 > Day 1 > Day 7 > Day 3; p = 0.015), and Acidimicrobiales (Day 1 > Day 3 > Day 14 > Day 7; p = 0.036) differed significantly in relative abundance between storage times. It should be noted that the Bradyrhizobiaceae is a family within Rhizobiales and Rubrobacteraceae is a family within Rubrobacterales. A single class, the Thermomicrobia, differed significantly between time treatments (Day 14 > Day 7 > Day 3 > Day 1; p = 0.037). Relative abundances of all taxa that differed significantly between temperature and time treatments are shown in the PCoA plots in figures S3, S4, and S5.

Relative abundances differed between storage times within samples stored at -20°C for the Planococcaceae (Day 7 > Day 14 > Day 1 > Day 3; p = 0.011) and the Haliangiaceae (Day 3 > Day 1 > Day 7 > Day 14; p = 0.036). The order Nitrososphaerales differed significantly in relative abundance between time treatments within the 4°C samples (Day 14 > Day 1 > Day 7 > Day 3; p = 0.031). The class Thaumarchaeota differed significantly in relative abundance between time treatments within 4°C samples (Day 14 > Day 1 > Day 7 > Day 3; p = 0.016).

### Correlations between relative abundance and storage time

Both the Alphaproteobacteria (r = 0.54, p = 0.028) and the Thermomicrobia (r = 0.54, p = 0.015) classes were significantly positively correlated with storage time. The relative abundance of the order Rhizobiales was also positively correlated with storage time (r = 0.60, p = 0.012). There was a significant negative correlation between the Haliangiaceae and storage time within samples stored at -20°C (r = -0.89, p = 0.018) and a positive correlation between the Hyphomicrobiaceae and storage time within samples stored at RT (r = 0.90, p = 0.011).

### Supervised learning classification

Supervised learning analysis, based on 10-fold cross-validation, failed to consistently distinguish between either time or temperature treatments. However, supervised learning easily distinguished between the 13 EMP studies represented here with an error of only 3.6% (±2.3%; random is 78%). All samples sequenced for the present study were accurately assigned with probabilities of 88% or greater.

### Interactions between time and temperature

The proportion of variance in the distances between communities explained by temperature and time treatments was nearly identical both when measured as marginal effects and when first conditioned on the variance explained by the other factor (Table S6), indicating that little if any interaction exists between these two storage variables. The proportion of variance explained by either variable, marginally or conditionally, was always under 3%. Despite potentially significant effects of these variables, their influence was still quite limited. In contrast, the proportion of variation in the EMP data explained by sample origin was 47%.

## Discussion

Overall, alpha diversity was significantly influenced by both storage temperature and storage time. The reasons for significantly higher alpha diversities on days three and seven are unclear, though may be a result of higher evenness on these days allowing for the detection of a greater number of rare taxa. It is likely that the members of the community continued to interact over time and, as each sample was quarantined from the others, caused the structure to change and the communities to diverge. The differences in the phylogenetic community comparison metric, UniFrac, within the three and seven day treatments mean that particular bacterial clades became extinct or fell below the limit of detection after only a few days of storage, but for which clades this happened first was unpredictable. This community decay likely occurs, to some extent, regardless of storage conditions.

The results for samples stored at different temperatures were clear. Alpha diversity was highest and differences between samples were lowest when samples were frozen, and the opposite applied when samples were stored at room temperature. Our results based on soil from a temperate climate suggest that best practices for storing soil samples for microbial analyses should include freezing, especially when fine-scale community resolution is needed for investigating the distribution of rare taxa. However, our data are based on soil from just a single site in a single climate. Communities and even specific taxa from different environments (e.g. tropical versus tundra soils) may respond differently to such conditions and other precautions may be required for preserving soil that normally experiences subzero temperatures in nature. Additional studies that include soil from a wide variety of climates would be highly useful.

Although no individual OTUs (97%) differed significantly in relative abundance between time treatments, three families, three orders, and a class of bacteria did. These differences are only detectable at higher taxonomic levels, showing that entire clades increase and decrease in aggregate over time, as was found for certain bacterial taxa in human fecal samples [21]. Despite the variations in relative abundance over time, correlations between time and taxon relative abundance were infrequent, indicating that relative abundances do not vary linearly with time but, rather, shift somewhat unexpectedly. Changes in the representation of large clades of bacteria also indicate that the metagenomic content of the community, not just 16S rRNA diversity, is almost certainly changing as well. A similarly designed study incorporating metagenome shotgun sequencing [32,33] – a more sensitive and information-rich method than the 16S rRNA sequencing implemented here – would yield useful additional insights.

While these results encourage consistency in sample storage and preparation, our comparisons with other EMP soil samples suggest that soil communities will, even after two weeks at room temperature, be far more similar to their original communities than to other soil samples, including those from similar biotic zones (Figure 3). And, although alpha diversities do shift within stored communities over time and temperature, the scale over which they shift is far lower than the range of soil alpha diversities across EMP data sets. However, the dissimilarity between the samples analyzed here and other EMP soils taken from around the world is not surprising and, while illustrating that soil communities do not change drastically over the times and temperatures examined, these differences tell us little about the degree of difference we might see between multiple samples taken from the same site. We take this as further indication that samples must be treated with consistency. Faithfully maintaining community structure is difficult over times and temperatures but consistency can prevent bias.

Lauber et al. [16] found that the impacts of different preservation methods on soil microbial communities were rather minor and that even samples preserved at different temperatures for different lengths of time can still be useful for community analysis. Our results largely support their findings; differences in preservation time and temperature are unlikely to drastically change community structure and composition. However, as technology allows for more resolved investigations of microbial environments, even very small alterations in the presence or relative abundance of taxa due to experimental treatments can significantly interfere with community comparisons. We must, therefore, be cautious; factors that we may typically not consider as influential may now drive observable differences between samples.

## Supporting Information

Figure S1 Principal coordinates plots based on weighted UniFrac distances showing similarities of samples between temperature and time treatments. (TIF)

Figure S2 Principal coordinates plots based on Bray-Curtis distances showing similarities of samples between temperature and time treatments. (TIF)

Figure S3 Principal coordinates plots based on unweighted UniFrac distances. Point sizes represent relative abundance of taxa indicated in each plot. Colors and shapes in (A) and (B) correspond to different temperature treatments and in (C) through (I) to different time treatments as in Figure 2.
(TIF)

Figure S4 As in Figure S3 except PCoA is based on weighted UniFrac distances. (TIF)

Figure S5 As in Figure S3 except PCoA is based on Bray-Curtis distances. (TIF)

Table S1 Sequencing throughput by sample. (XLS)

Table S2 T-test results of differences in alpha diversity between treatments. (XLS)

Table S3 Ranges of alpha diversities for samples from all EMP studies examined here. (XLS)

Table S4 ANOSIM and RDA comparisons of beta diversity of all combinations of treatments. (XLS)

Table S5 T-test results of differences in within treatment beta diversity. (XLS)

Table S6 Marginal and conditional proportions of variance in current study sample communities explained by storage time and temperature. (XLS)

Table S7 All EMP samples utilized in this study. (XLS)
