# Supplementary figures and images for: Investigating the Impact of Storage Conditions on Microbial Community Composition in Soil Samples
Source: PLoS One. 2013 Jul 31;8(7):e70460. doi: 10.1371/journal.pone.0070460 (PMC3729949; doi:10.1371/journal.pone.0070460)

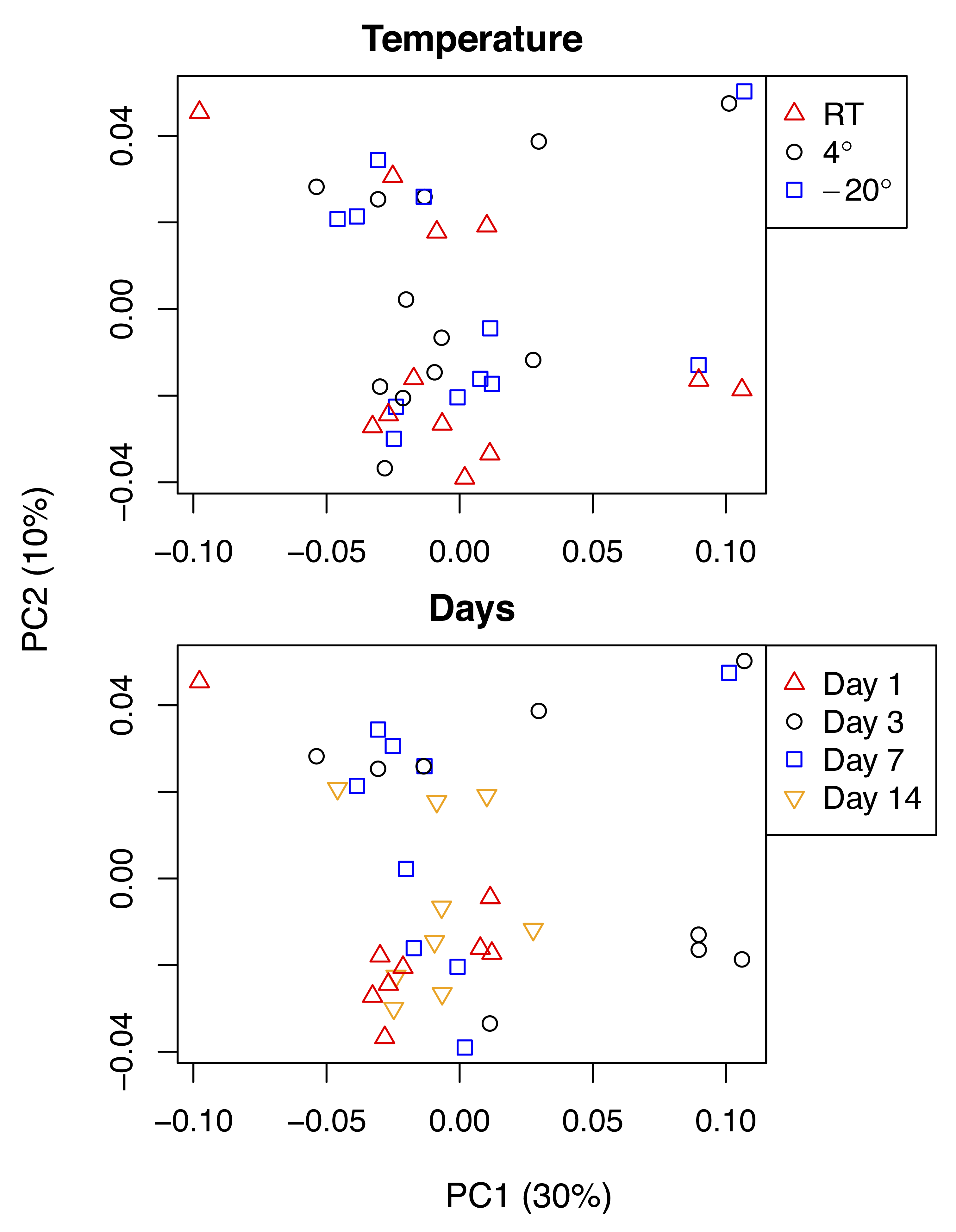

Supplement: Figure S1 — (TIF) [file pone.0070460.s001.tif]

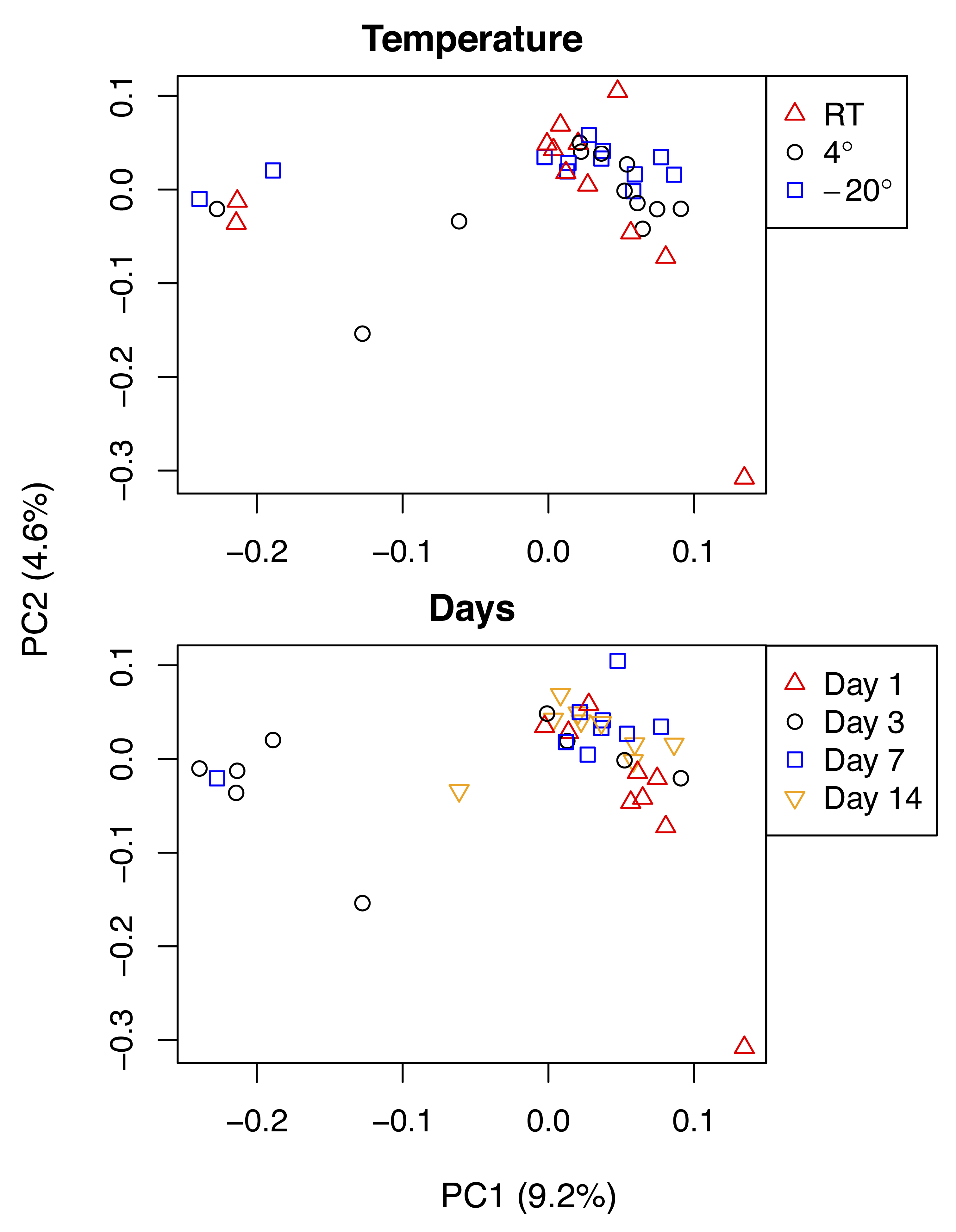

Supplement: Figure S2 — (TIF) [file pone.0070460.s002.tif]

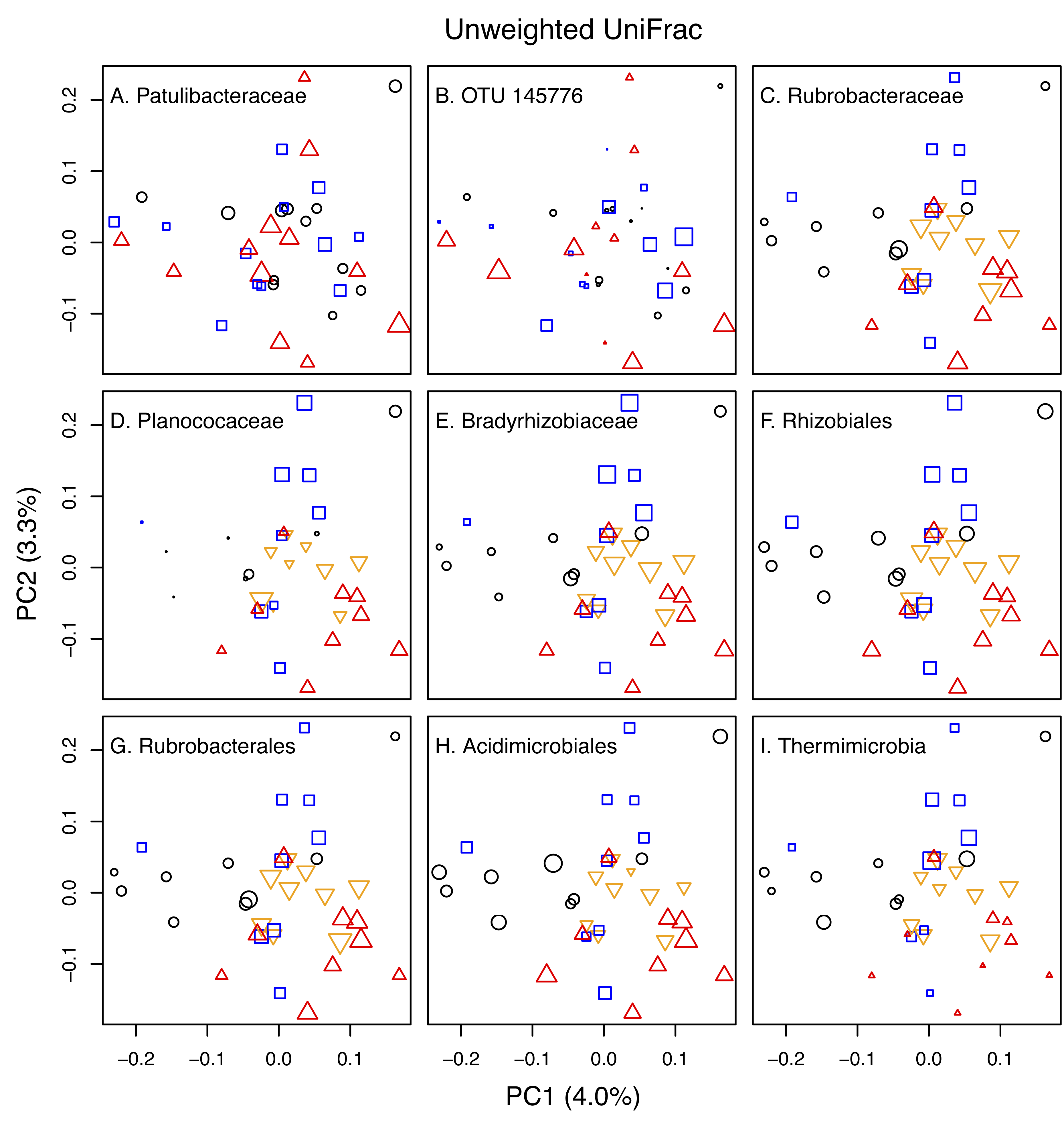

Supplement: Figure S3 — Colors and shapes in (A) and (B) correspond to different temperature treatments and in (C) through (I) to different time treatments as in Figure 2. (TIF) [file pone.0070460.s003.tif]

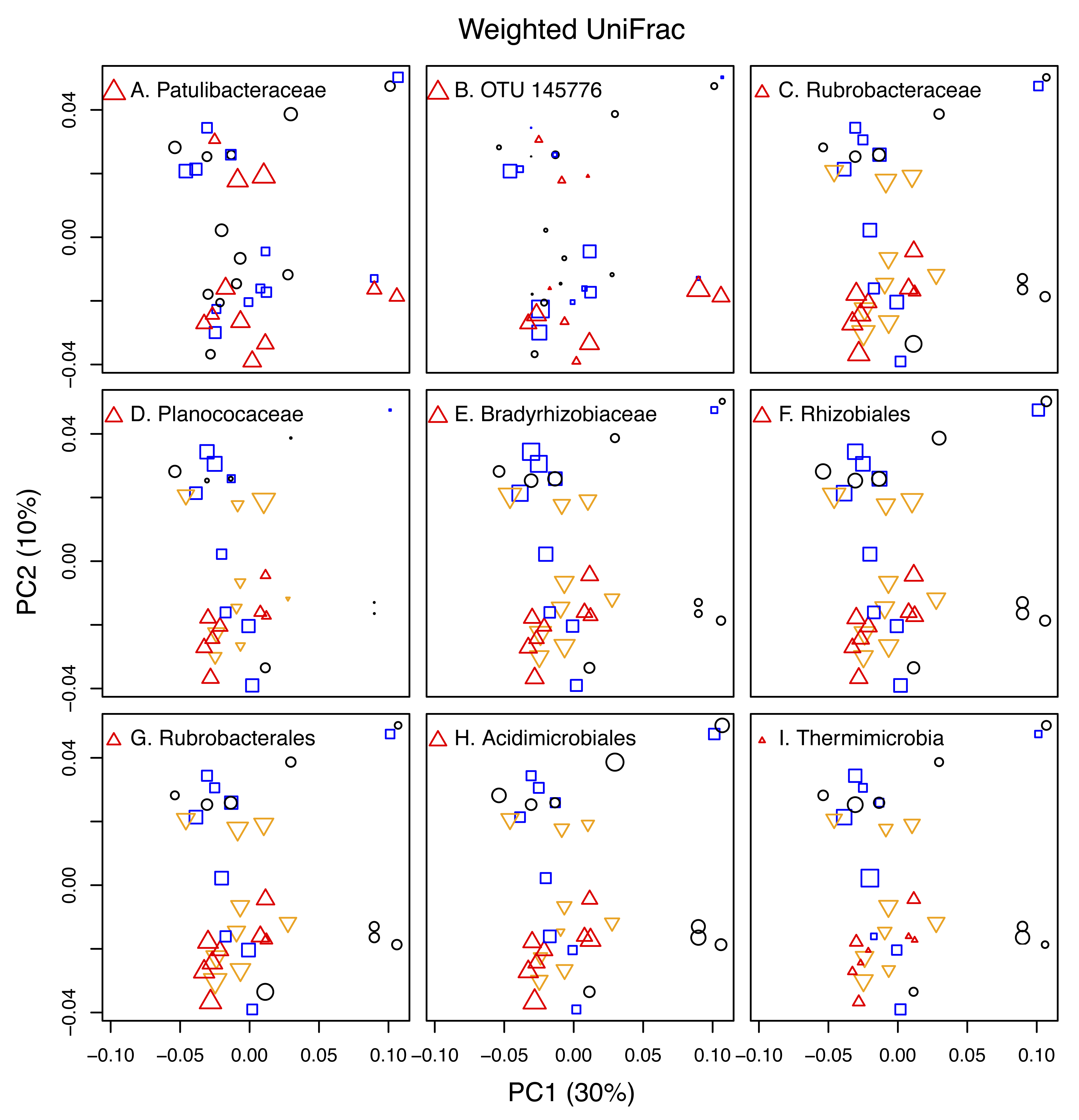

Supplement: Figure S4 — (TIF) [file pone.0070460.s004.tif]

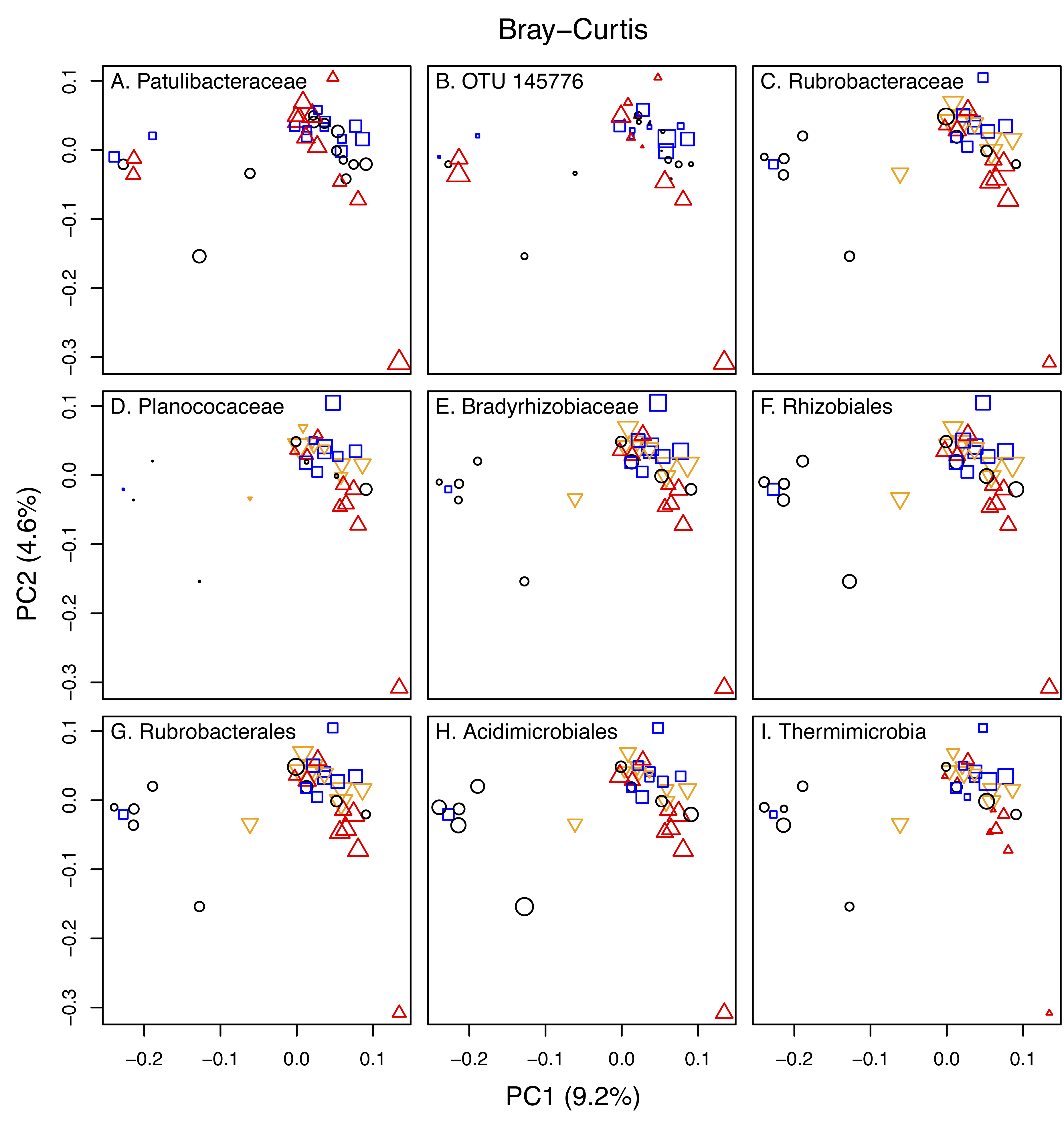

Supplement: Figure S5 — (TIF) [file pone.0070460.s005.tif]
